# Supplementary figures and images for: Association of visceral adiposity index and lipid accumulation products with prediabetes in US adults from NHANES 2007–2020: A cross-sectional study
Source: PLoS One. 2024 Sep 30;19(9):e0311312. doi: 10.1371/journal.pone.0311312 (PMC11441703; doi:10.1371/journal.pone.0311312)

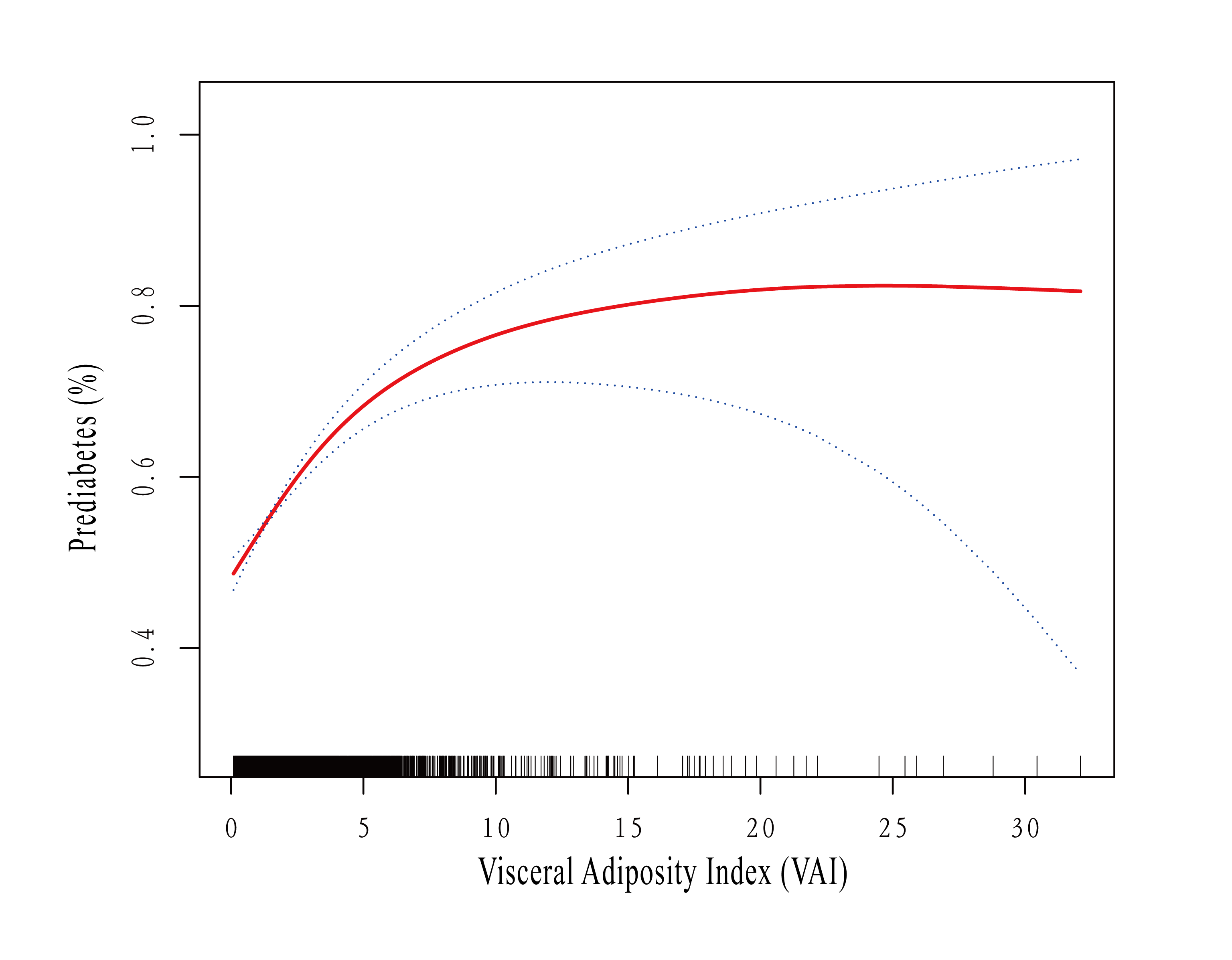

Supplement: S1 Fig — (TIF) [file pone.0311312.s001.tif]

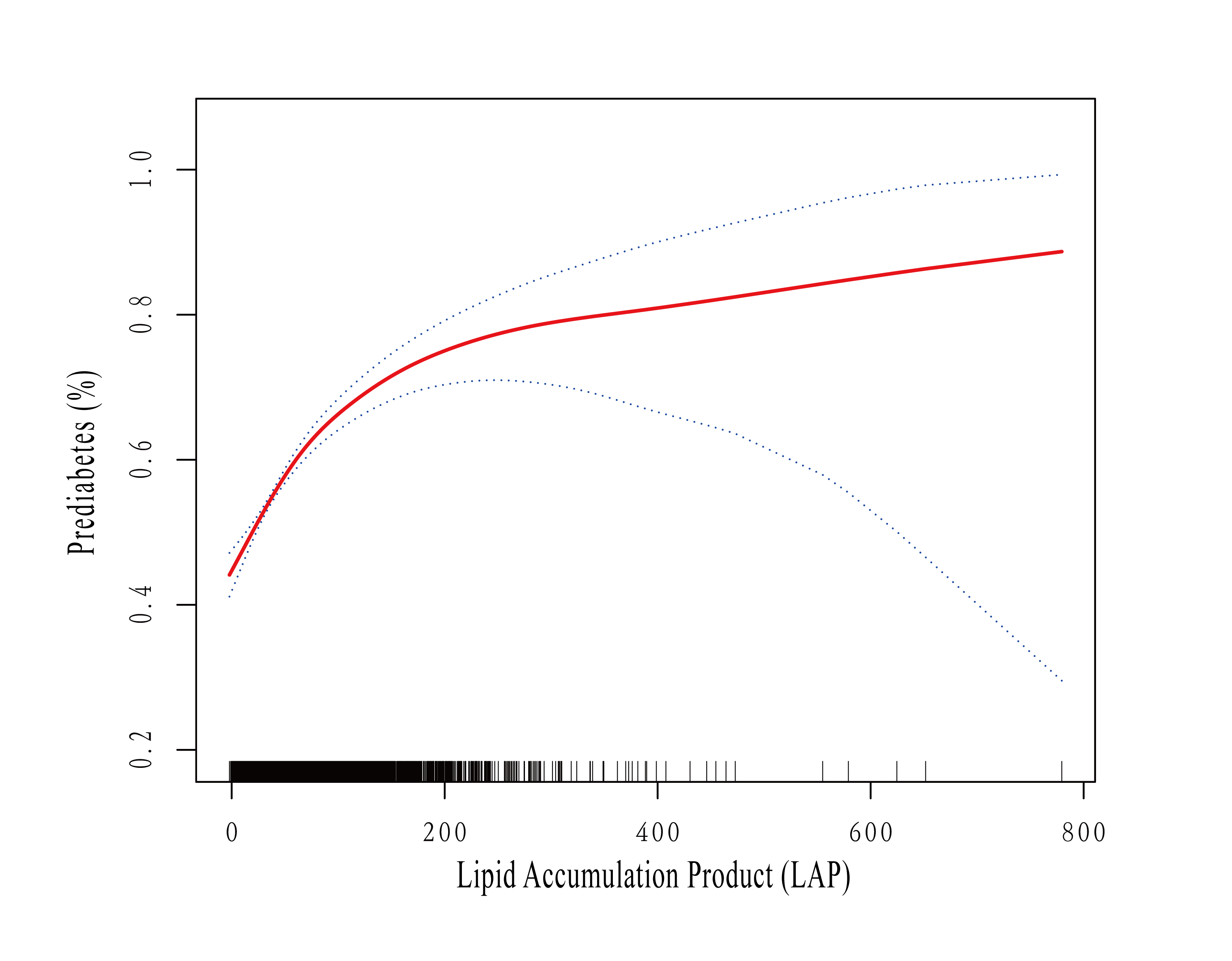

Supplement: S2 Fig — (TIF) [file pone.0311312.s002.tif]
