# Supplementary material for: Association of visceral adiposity index and lipid accumulation products with prediabetes in US adults from NHANES 2007–2020: A cross-sectional study
Source: PLoS One. 2024 Sep 30;19(9):e0311312. doi: 10.1371/journal.pone.0311312 (PMC11441703; doi:10.1371/journal.pone.0311312)
Supplement: Attachment — Submitted filename: PONE_comments.docx [file pone.0311312.s003.docx]

Comments:

The introduction does not include the research conducted with VAI and LAP on ethnicities other than South Asian population. The objective is stated but is deficient by hypothesis.

Methodology:

The study design is not mentioned and the sample size estimation using multistage probability sampling requires description. I understand the data is collected by NHANES. How are the anthropometric measurements recorded and We assume they follow standard guidelines but this requires a mention.

The covariates data such as social, demographics, caloric intake, physical activity, smoking and alcohol history, were they all available in the database or these information were later recorded from participants. This can make a difference because the biochemical and body composition measurements must match with the timing when history is collected from participants.

Statistical analysis:

The regression analysis adopted requires explanation stepwise. Was the multivariate analysis conducted based on the results from univariate analysis? Three models were adopted for multivariate analysis- this is mentioned later in the results but not in methodology. Why is the OR mentioned only once for three models of multivariate analysis (line 226 & 228). The table show the OR for all three models.

Table 3, first column, What is the meaning of reference? This requires description.

Table 4 mentions inflection point, but the determination of inflection point needs to be mentioned.
